# Analysis of PR-1, PR-2, PR-10, chitinases and chitinase-like proteins genes expression in pea roots under the action of salicylic acid and methyl jasmonate

**DOI:** 10.18699/vjgb-26-24

**Published:** 2026-04

**Authors:** A.M. Egorova

**Affiliations:** Kazan Institute of Biochemistry and Biophysics of Kazan Scientific Center of the Russian Academy of Sciences, Kazan, Russia

**Keywords:** marker gene, PR proteins, signaling, salicylic acid, jasmonic acid, chitinase-like proteins, phytoimmunity, маркерный ген, PR-белки, сигналинг, салициловая кислота, жасмоновая кислота, хитиназа-подобные белки, фитоиммунитет

## Abstract

Stress phytohormones – salicylic and jasmonic acids – participate in the plant defense response. The increase in the content of these compounds during plant infection by pathogens leads to the activation of signaling pathways, ultimately resulting in changes in gene expression and protein synthesis, including a group of pathogenesis-related (PR) proteins. Phytohormone-dependent so-called marker genes, are used to assess the activation of these signaling pathways. In this study, PR-1 genes, which are markers for salicylic acid signaling and expressed in pea roots, were identified and characterized, and the effects of salicylic acid and methyl jasmonate on their expression were analyzed. It was shown that in pea roots, PR-1, encoded by the Psat1g156240 gene, is among the most significantly expressed genes in the control. Salicylic acid did not cause a change in the expression of this gene; however, it was induced by methyl jasmonate after 24 h. Analysis of the expression of other genes encoding PR-1 proteins showed that salicylate had no effect on their expression after 24 and 72 h. Analysis of the expression of genes encoding chitinases and chitinase-like proteins showed that the former do not exhibit specificity in response to the salicylate and methyl jasmonate, except for the Psat1g131280, the expression of which increased after 24 and 72 h of treatment with methyl jasmonate. Genes Psat1g147600, Psat1g147560, Psat1g149120 encoding chitinase-like proteins, were barely expressed in pea roots in control, and were specifically induced by salicylic acid. β-1,3-glucanase genes were not induced in roots by the studied phytohormones. The obtained results allowed to reveal specific genes including chitinase-like proteins, the expression of which is salicylate-inducible. These genes can be used for the assessment of the activation of the salicylate-dependent signaling pathway in pea roots.

## Abstract (ru)

В реализации защитного ответа растений принимают участие стрессовые фитогормоны – салициловая и жасмоновая кислоты. Повышение содержания этих соединений при инфицировании растений патогенами приводит к активации сигнальных путей, в конечном итоге изменяющих экспрессию генов и синтез белков, среди которых выделяется группа индуцируемых, связанных с патогенезом белков (PR). Для оценки активации этих сигнальных путей используются фитогормон-зависимые, так называемые маркерные гены. В настоящей работе выявлены и охарактеризованы экспрессирующиеся в корнях гороха гены маркерных для салицилатного сигналинга белков PR-1. Проанализировано влияние салициловой кислоты и метилжасмоната на их экспрессию. Показано, что в корнях гороха ген Psat1g156240, кодирующий PR-1, входит в число наиболее значительно экспрессирующихся генов. Салициловая кислота не вызывала изменение экспрессии этого гена, в то же время он индуцировался метилжасмонатом через 24 ч. Анализ экспрессии других генов, кодирующих белки PR-1, показал, что салицилат не влиял на их экспрессию через 24 и 72 ч. При анализе экспрессии генов хитиназ и хитиназа-подобных белков первые не проявляли специфичность ответа на действие салицилата и метилжасмоната, за исключением гена Psat1g131280, экспрессия которого повышалась через 24 и 72 ч при действии метилжасмоната. Гены хитиназа-подобных белков, Psat1g147600, Psat1g147560, Psat1g149120, практически не экспрессировались в корнях гороха контрольного варианта и специфично индуцировались салициловой кислотой. Гены β-1,3-глюканаз не индуцировались исследованными фитогормонами в корнях. Полученные результаты позволили определить конкретные гены, кодирующие хитиназа-подобные белки, экспрессия которых индуцируется салициловой кислотой. Эти гены могут быть использованы для оценки активации салицилат-зависимого сигнального пути в корнях гороха.

## Introduction

Throughout their lifestyle, plants are constantly exposed to pathogens and unfavorable environmental conditions. Phytoimmunity enables plants to cope with pathogen attacks, overcome these unfavorable conditions, and survive. Furthermore, plants have evolved a wide array of defense mechanisms in response to stressors and pathogen attacks. These include pathogen-specific resistance genes, antimicrobial compounds such as phytoalexins, and pathogen-induced defense proteins (de Wit, 2007).

Salicylic acid (SA) and jasmonic acid (JA) are key phytoimmunity factors involved in the realization of plant defense responses (Ding P., Ding Y., 2020; Wang et al., 2021). SA is generally associated with defense against biotrophic and hemibiotrophic pathogens, which feed on living plant tissues, whereas JA is primarily involved in defense against necrotrophic pathogens and herbivorous insects (Glazebrook, 2005). Pathogen attack on plants leads to the production of signaling molecules and the activation of signaling pathways involving one of the immunity factors mentioned above (Ding L.N. et al., 2022). Activation of these signaling cascades alters gene expression and protein synthesis. These proteins either have direct antimicrobial properties or are involved in the synthesis of defense compounds.When studying plant responses to various factors, it is often necessary to assess the activation of specific signaling pathways to understand the mechanisms underlying the response. This is most commonly achieved by analyzing the activation of expression of so-called marker genes. These genes have primarily been characterized in model plants such as Arabidopsis, tobacco, and rice, and mainly in aboveground organs. Nevertheless, results obtained from model plants are not always relevant to other plant species, particularly to significant agricultural crops. For example, research on Brassica rapa has shown that widely used marker genes for salicylate- and jasmonate/ethylene-dependent signaling pathways lack specificity in their expression in response to these hormones, and their expression levels differ between shoots and roots (Papadopoulou et al., 2018). In another study on Capsicum annuum, the PR-1 gene, a marker for salicylate signaling, was found to be a more suitable candidate for the JA/ethylene-dependent pathway rather than the SA pathway (Perez‐Aranda et al., 2024). In Brachypodium distachyon, analysis of BdPR1-4 and BdPR1-5 gene expression revealed that they are regulated by both SA and JA (Kouzai et al., 2016).

In the post-genomic era, advances in omics technologies enable the generation and analysis of large datasets, including gene expression and protein synthesis data. The available pool of transcriptomic data allows for the analysis of the full spectrum of genes expressed in a specific organ, both constitutive ones and those altered under various conditions. The identification and characterization of such genes, which play a role in plant adaptation, can be useful for breeding of resistant cultivars (Tyagi et al., 2022).

Pathogen-induced proteins with antipathogenic activity (Pathogenesis-Related or PR proteins) play a crucial role in the realization of plant defense responses. Based on their functional characteristics, they are classified into 17 classes. Many PR proteins are induced not only by pathogens but also by stress-related phytohormones (van Loon et al., 2006). Salicylate-dependent genes are important in mediating plant defense responses (Maleck et al., 2000; Blanco et al., 2009). One of the most well-known marker genes for salicylatedependent signaling is PR-1 (Breen et al., 2017). Alongside with PR-1, other members of the PR gene family – such as β-1,3-glucanases (PR-2) (Hennig et al., 1993; Jayaraj et al., 2004) and chitinases (PR-3, 4, 8, 11) (Davis et al., 2002; Tarchevsky et al., 2010) – are also regulated by SA.

In recent years, significant attention has been paid to the processes occurring in the rhizosphere and to the interactions between plant roots and pathogens, symbiotic bacteria, and other soil inhabitants. The crop yield also depends on these relationships, since infection of roots with pathogens and the efficiency of symbiosis formation with nodule bacteria (for legumes) significantly impact plant growth and yield (Solomon et al., 2024). Furthermore, roots play a crucial role in plant life not only as “providers” of water and minerals but also as active participants in defense responses. Certain compounds with antipathogenic properties, such as alkaloids and terpenoids, are synthesized in the roots and transported to aboveground organs. In addition to this, roots implement their own specific defense mechanisms (Erb, 2012).

Previously, in our work, we observed a difference in the proteomic response of pea leaves and roots to exogenous SA application, which may also reflect a difference in their defense responses. In pea leaves, SA upregulates the content of several PR proteins – chitinases and β-1,3-glucanases (Tarchevsky et al., 2010) – whereas in the roots, the content of chitinase-like proteins belonging to glycoside hydrolase family 18 (GH18) increased the most significantly (Egorova, 2025). Obviously the differing environmental conditions of aerial organs and roots effect the specifics of their responses to various stimuli. Therefore, identifying the characteristics of root responses, including those to pathogen attacks, is important as the data can be utilized in breeding cultivars resistant to soil-borne pathogens and in developing agrobiotechnology approaches to protect specific crops from soil pathogens. When studying the features of root defense responses and assessing the activation of specific signaling pathways, the use of adequate marker genes is essential. In this context, the search for, characterization of, and understanding of the expression patterns and specificity of these genes in response to particular signaling compounds represents an important task.

In this study, we identified and characterized genes encoding PR-1, chitinases and chitinase-like proteins, β-1,3-glucanases, and PR-10 in the Pisum sativum roots. We analyzed the expression of these genes in the control and in response to SA and methyl jasmonate (MeJA). Gene expression was assessed using comparative transcriptomic analysis of control, SA-treated, and MeJA-treated pea roots

## Materials and methods

Plant growth and treatment. Pea (P. sativum L.) seeds (cultivar Tan) were surface-sterilized in 0.05 % potassium permanganate solution for 20 minutes and then thoroughly rinsed with distilled water. The seeds were germinated on moist filter paper in the dark for 3 days. The seedlings were grown in ¼-strength Hoagland–Arnon liquid medium under a 16-hour photoperiod at 25 °C. Eight-day-old seedlings were used for subsequent experiments. For the experimental treatments, 50 μM SA (Sigma, USA) or 20 μM MeJA (Serva, Germany) was added to the growth medium. The compounds were first dissolved in 500 μl of ethanol. The control group consisted of seedlings grown on ¼-strength Hoagland–Arnon solution supplemented with a corresponding volume of ethanol, calculated to account for dilution. Pea roots were harvested and frozen in liquid nitrogen at 24 and 72 h posttreatment.

Transcriptome analysis. Total RNA was isolated from 200 mg of pea roots using the ExtractRNA reagent (Evrogen, Russia) according to the manufacturer’s protocol. RNA concentration and quality were assessed using an Implen Nano- Photometer (Implen, GmbH) and by 1 % agarose gel electrophoresis. The preparation of cDNA libraries, high-throughput sequencing, and bioinformatic analysis of the transcriptomic data were performed by Novogene (Beijing, China).RNA quality was verified using the Agilent Nano 6000 Assay Kit of the Bioanalyzer 2100 system (Agilent Technologies, CA, USA). mRNA was enriched from total RNA using poly-T oligo-attached magnetic beads. Fragmentation was carried out using divalent cations under elevated temperature in First Strand Synthesis Reaction Buffer (X5) (Thermo Fisher, USA). First-strand cDNA was synthesized using random hexamer primers and M-MuLV Reverse Transcriptase (Thermo Fisher, USA). Second-strand cDNA synthesis was subsequently performed using DNA Polymerase I and RNase H. After adenylation of the 3′ ends of the DNA fragments, Adaptor with hairpin loop structure was ligated to prepare for hybridization. In order to select cDNA fragments preferentially 370–420 bp in length, the library fragments were purified with the AMPure XP system (Beckman Coulter, USA). PCR products were purified (AMPure XP system) and library quality was assessed on the Agilent Bioanalyzer 2100 system (Agilent, USA).

After clusters were generated on a cBot Cluster Generation System using the TruSeq PE Cluster Kit v3-cBot-HSv3-cBot-HS (Illumina, USA), the library preparations were sequenced on an Illumina NovaSeq 6000 platform (Illumina, USA), generating 150 bp paired-end reads. Raw data were processed using Perl scripts. At this stage, reads containing adapters, poly-N sequences, and low-quality reads were removed to obtain clean data. Quality metrics Q20, Q30, and GC content were calculated. All downstream analyses were based on these high-quality clean data. The P. sativum_v1a genome (Kreplak et al., 2019) was used as the reference (https://urgi.versailles. inra.fr/Species/Pisum). The reference genome was indexed, and the clean reads were aligned to it using Hisat2 v2.0.5.

The number of reads mapped to each gene was counted using feature Counts v1.5.0-p3. The FPKM (Fragments Per Kilobase of transcript sequence per Millions base pairs sequenced) for each gene was calculated based on the gene length and the mapped read count. FPKM simultaneously accounts for the effects of sequencing depth and gene length on the read count (Trapnell et al., 2010).

Differential gene expression analysis was performed using the DESeq2 R package (1.20.0) (Love et al., 2014). The resulting p-values were adjusted using the Benjamini and Hochberg approach for controlling the false discovery rate. Genes with an adjusted p-value ≤ 0.05 and an absolute log2 fold change ≥1 were considered differentially expressed. A minimum of three biological replicates was used for each condition.

In this study, gene expression levels are presented as FPKM values, as not all analyzed genes were differentially expressed. Figures display FPKM values with standard deviations. Differentially expressed genes are indicated in the figures with an asterisk (*). The sequencing data have been deposited in the NCBI SRA (BioProject PRJNA1358375).

Bioinformatic analysis. BLAST searches for predicted pea genes and protein sequences were performed against the P. sativum genome_v.1a (Kreplak et al., 2019) in the Ensemble- Plants database (https://plants.ensembl.org/Pisum_sativum/ Info/Index). Multiple alignment of protein sequences was conducted using the MEGA v.10.2.6 software (Kumar et al., 2018). Full-length amino acid sequences for multiple alignments were retrieved from the UniProt and NCBI databases. Protein domain organization was analyzed and predicted using the Conserved Domains Database (https://www.ncbi.nlm.nih. gov/Structure/cdd/wrpsb.cgi).

## Results

A transcriptomic analysis of pea seedling roots of control plants and those treated with SA (24 and 72 h) and MeJA (24 and 72 h) was performed. Each sample contains between 36 and 57 million reads. The quantity, quality, and characteristics of the obtained data are presented in the Supplementary Material1

Supplementary Materials are available in the online version of the paper: https://vavilovj-icg.ru/download/pict-2026-30/appx15.xlsx


Alignment of the reads to the reference pea genome (P. sativum_ v1a) (Kreplak et al., 2019) identified approximately 20 thousand transcripts. After 24 h, SA altered the expression of 1,476 genes, of which 635 were upregulated and 841 were downregulated. After 72 h of SA treatment, the expression of 2,420 genes was altered, with 977 upregulated and 1,443 downregulated. MeJA treatment for 24 h changed the expression of 3,384 genes (1,546 upregulated, 1,838 downregulated). After 72 h, MeJA altered the expression of 5,462 genes, with 2,218 upregulated and 3,244 downregulated ones.

This study analyzed the expression of PR-1, chitinase and chitinase-like protein, β-1,3-glucanase, and PR-10 genes in pea roots to assess their potential as markers for salicylic acid signaling and to identify specific isoforms induced specifically by SA. These gene groups were selected because PR-1, chitinases, β-1,3-glucanases, and PR-10 have been shown to be induced by SA (Davis et al., 2002; Li et al., 2005; Spoel et al., 2007; Xu et al., 2024) and to possess antipathogenic activity (van Loon et al., 2006), while chitinase-like proteins are significantly induced by SA in pea roots (Egorova, 2025). The expression of the identified genes was evaluated under the influence of both SA and MeJA, as both hormones are involved in plant defense responses and determine the features of the implemented defense strategy

**Identification and expression analysis of PR-1 genes in pea roots**

**Fig. 1.**
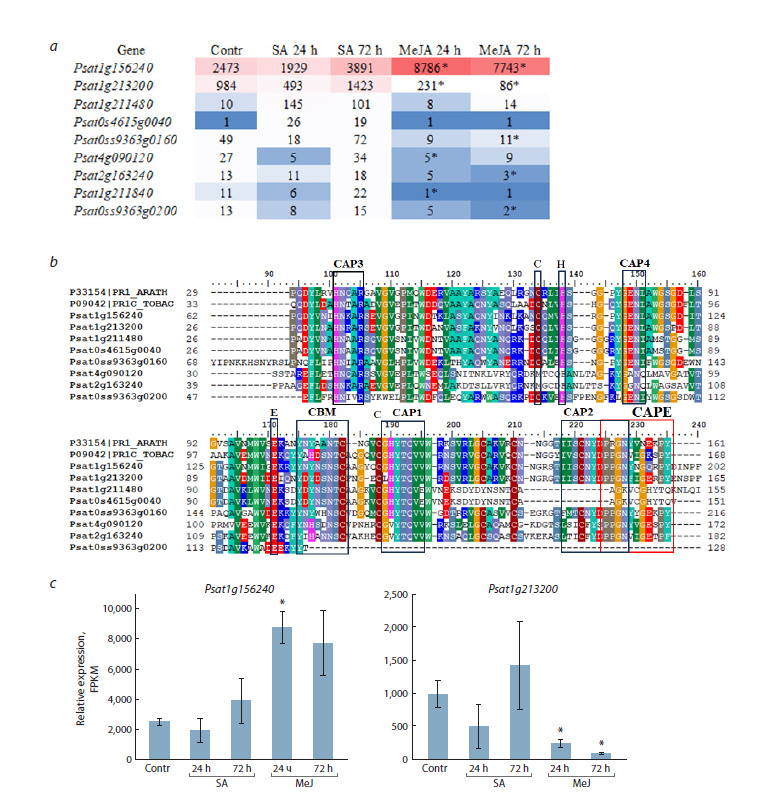
Cysteine-rich secretory proteins whose genes are expressed in pea roots. a – list of CAP proteins (PF 00188) expressed in pea roots in the control (Contr) and under the action of SA (50 μM) and MeJA (20 μM) for 24 and 72 h. The color scale represents highly expressed (red) and weakly expressed (blue) genes; the expression level is shown in FPKM; b – multiple sequence alignment of PR-1 proteins from Arabidopsis (P33154), tobacco (P09042), and CAP proteins of pea roots. Black rectangles indicate conserved CAP1–CAP4 domains, conserved cysteine, histidine, glutamic acid residues, and the caveolin-binding motif (CBM) responsible for sterol binding. The CAPE peptide is highlighted in red; c – differentially expressed CAP genes in pea roots under SA and MeJA treatment. Here and in Figures 2–4: data represent ± SD mean values of three to five biological replicates. * Indicates significant differences in gene expression in SA- and MeJA-treated samples compared to the control (p < 0.05 and |log2 FC| ≥ 1).

PR-1 belongs to the family of cysteine-rich secretory proteins (CAPs), and pea genome contains 12 sequences putatively assigned to this family. A BLAST search in the pea genomic database using characterized PR-1 sequences from tobacco (P09042) and Arabidopsis (AT2G14610) revealed similar sequences in pea. Analysis of the expression of the revealed PR-1 genes in the pea genome showed that nine CAP genes are expressed in the control roots (Fig. 1a). The expression of genes Psat7g104840, Psat6g069320, and Psat1g21192 was low (<10 FPKM), and they are not discussed further in this work.

To characterize the pea proteins, a multiple alignment of the amino acid sequences of PR-1s, encoded by genes expressed in roots, with annotated proteins from Arabidopsis and tobacco, was performed. The protein encoded by the most highly expressed pea roots gene, Psat1g156240, shares 60 % amino acid sequence identity with the PR-1 protein P09042 from Nicotiana tabacum and with AtPR1 (AT2G14610) from Arabidopsis. The protein encoded by the gene Psat1g213200 shares 66.4 % identity with PR1A_TOBAC (P08299) and 65 % with AtPR1. CAP proteins are characterized by the presence of conserved CAP motifs (CAP1– CAP 4) and a CAPE peptide (Han et al., 2023). The two PR-1 proteins encoded by genes Psat1g211480 and Psat0s4615g0040 have a truncated CAPE peptide and lack the CAP2 motif (Fig. 1b), while other conserved motifs are present in their sequences.In the control, the expression of Psat1g156240 and Psat1g213200 was the highest compared to other PR-1 genes. The effect of SA and MeJA on the expression of PR-1 genes was assessed (Fig. 1a). The analysis showed that Psat1g156240 is upregulated by MeJA at 24 h. SA did not cause a significant change in the expression of this gene. The expression of the Psat1g213200 gene was not changed significantly by SA but decreased under the action of MeJA (Fig. 1c). The expression levels of the Psat1g211480 and Psat0s4615g0040 genes in the control were lower compared to the two previous genes and did not change in response to either SA or MeJA at the studied time points. The expression of other CAP genes – Psat0ss9363g0160, Psat4g090120, Psat2g163240, Psat1g211840, and Psat0ss9363g0200 – was low in the control, did not change under the action of SA, but was downregulated by MeJA (Fig. 1a).

**Identification and analysis of chitinase and chitinase-like protein gene expression in pea roots**

Chitinases (E.C. 3.2.1.14) belong to glycosyl hydrolase families 18 (GH18) and 19 (GH19). The chitinase genes Psat3g016800, Psat1g084840, Psat2g145680, Psat3g016400, Psat5g291720, Psat2g128240, Psat4g175600, Psat4g175720, Psat0s794g0040, Psat5g256760, Psat5g298520, Psat1g169040, Psat4g171240, Psat4g177760, Psat5g020240, Psat6g166120, Psat6g194800 are either not expressed in roots or their expression level is low (<10 FPKM) and are therefore not discussed further in this work (see the Supplementary Material)

**Fig. 2.**
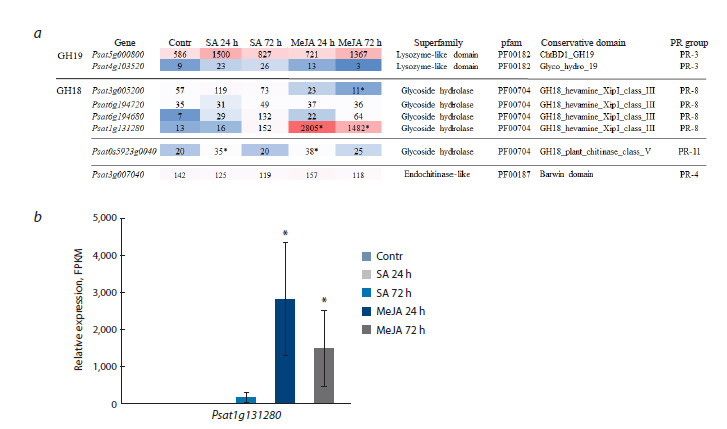
Expression of chitinase-like protein genes in pea roots under the action of SA (50 μM) and MeJA (20 μM) for 24 and 72 h (FPKM).

Transcriptomic data analysis revealed that six chitinase genes encoding proteins from the GH18 family and two genes from the GH19 family are expressed in pea roots, which in turn belong to different PR protein groups (Fig. 2a). All GH19 chitinases expressed in pea roots contain a lysozyme-like domain, including the most highly expressed gene Psat5g000800 (Fig. 2a). SA and MeJA did not cause a significant change in the expression of this gene. The expression of the second GH19 chitinase gene, Psat4g103520, which was significantly lower than that of the first gene, also did not change under the action of SA or MeJA.

The expression of genes encoding GH18 family proteins in the control was lower compared to the GH19 genes. Chitinases belonging to PR-3 contain a hevein-like domain. The expression of the genes Psat3g005200 and Psat6g194680, belonging to acidic endochitinases, did not change under salicylate treatment, but the expression of Psat3g005200 decreased under the action of MeJA after 72 h. The expression of Psat6g194680 did not change under SA or MeJA treatment (Fig. 2a). The expression of Psat1g131280, annotated as hevamine A, increased thousands of times under the action of MeJA after 24 and 72 h (Fig. 2). The expression of Psat0s5923g0040, also belonging to GH18, was slightly increased by SA and MeJA after 24 h (Fig. 2a).

Another member of the GH18 protein family, the product of the gene Psat3g007040, contains a barwin domain in addition to the chitin-binding domain. Proteins with this domain are classified as PR-4 proteins. Neither SA nor MeJA influenced the expression of this pea chitinase representative (Fig. 2a).

**Fig. 3.**
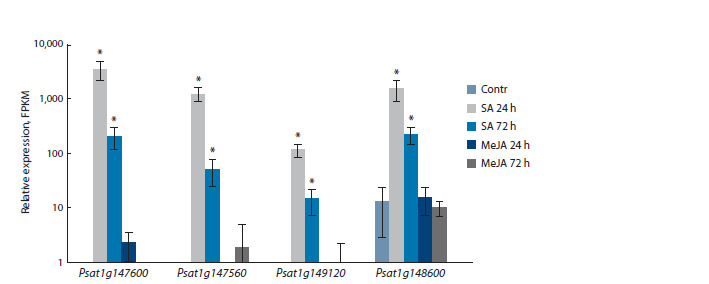
Expression of chitinase-like protein genes in pea roots under the action of SA (50 μM) and MeJA (20 μM) for 24 and 72 h (FPKM).

In the previous study (Egorova, 2025), significant induction of a group of proteins belonging to the GH18 family in pea roots by salicylate was shown. The identified proteins are encoded by the genes Psat1g147560, Psat1g147600, Psat1g149120 and Psat1g148600. Analysis of the chitinase activity of these proteins revealed that they do not degrade chitin and are thus classified as chitinase-like proteins (Egorova, 2025). A previous transcriptomic analysis of the effect of SA on these genes showed that the expression of all four genes was significantly increased by SA treatment, while being low in the control (Egorova, 2025). In the present work, we analyzed the influence of MeJA, which had no effect on the expression of any of the four genes studied (Fig. 3).

**Identification and analysis of PR-2 and PR-10 gene expression in pea roots**

Our previous work demonstrated the induction of β-1,3- glucanases (PR-2) and a disease resistance response protein (PR-10) in pea leaves by SA (Tarchevsky et al., 2010). A proteomic analysis of SA-treated pea root proteins showed an increased abundance of disease resistance response proteins and ABA-responsive proteins belonging to the PR-10 group, but we did not detect changes in the content of β-1,3-glucanases (Tarchevsky et al., 2010). The pea genome was searched for genes encoding these proteins, and their expression was analyzed based on transcriptomic data. In this part of the study, we focused on the most highly expressed genes in pea roots (>100 FPKM), as genes with lower expression levels did not show significant changes in response to phytohormone treatment (see the Supplementary Material).

**Fig. 4.**
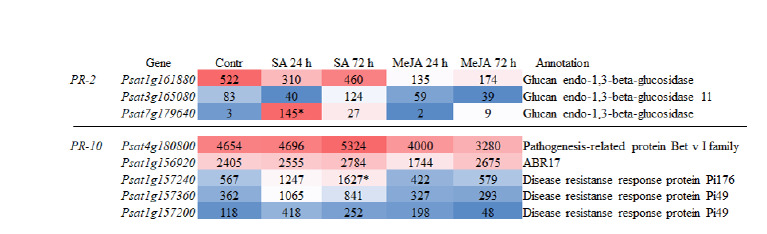
List of some most highly expressed (>100 FPKM) PR-2 (β-1,3-glucanase) and PR-10 genes in pea roots in the control (Contr) and under the action of SA (50 μM) and MeJA (20 μM) for 24 and 72 h. The color scale represents highly expressed (red) and weakly expressed (blue) genes; expression level is shown in FPKM.

More than 50 genes encoding β-1,3-glucanases were identified in the pea genome, of which approximately 20 were expressed in roots at varying levels (see the Supplementary Material). Psat1g161880 was highly expressed in the control, and its expression did not change in response to SA or MeJA (Fig. 4). The expression of Psat3g165080 was independent of SA and MeJA. The gene Psat7g179640 was practically not expressed in the control but was activated by SA after 24 h (Fig. 4).

More than 10 genes encoding PR-10 proteins are expressed in pea roots. One of the representatives of this group, encoded by Psat4g180800, was the most highly expressed gene in pea roots. Neither SA nor MeJA significantly affected the expression of this gene. The Psat1g156920, the product of which is annotated as an ABA-responsive protein 17, was also highly expressed, and its expression was not altered by the studied phytohormones. After 72 h, SA upregulated expression of Psat1g157240, the product of which is annotated as a disease resistance response protein (Fig. 4).

## Discussion

When working with non-model plants, which include the majority of agricultural species, discrepancies with results obtained on model plants arise. In particular, data on marker genes, the activation of expression of which is associated with the onset of specific phytohormone-dependent signaling pathways, were established using model plants like Arabidopsis and tobacco. This is important in studying plant defense mechanisms, as the findings can be applied to develop defense strategies for these species and in the breeding of resistant cultivars.

The main object of our research is pea, and we have previously studied the effect of phytoimmunity factors – salicylic and jasmonic acids – on the pea roots protein spectra, identifying the most significant changes in protein content (Tarchevsky et al., 2010; Yakovleva et al., 2013; Egorova, 2025). This study analyzed the expression of certain classes of PR genes and chitinase-like protein genes to assess their response to salicylate and MeJA, since we were faced with the need to choose adequate marker genes to evaluate the activation of the SA-dependent pathway in pea roots. The hormone treatment time points were based on our prior proteomic studies showing changes in the protein spectra at 72 h, and our interest in early events following the activation of phytohormone-dependent defense mechanisms.

First, we analyzed the expression of the widely accepted SA-dependent marker gene PR-1 and its paralogs in pea roots. The PR-1 protein was first isolated from TMV-infected tobacco leaves and could constitute up to 2 % of total protein in infected leaves (Alexander et al., 1993). However, not all PR-1 protein isoforms were induced upon infection. Early studies showed that PR-1 proteins participate in plant growth and developmental processes unrelated to pathogenesis – they accumulate in senescing plants leaves (Fraser, 1981) and in sepals of developing flowers (Lotan et al., 1989). Several studies demonstrated that overexpression of PR-1 enhances resistance to fungi (Sarowar et al., 2005) and bacteria (Kiba et al., 2007), with transgenic plants showing increased pathogen resistance (Ali et al., 2018). The protective functions of PR-1 proteins may be linked to their ability to interact with sterols and the presence of the CAPE peptide in their structure (Breen et al., 2017; Han, 2023). The CAPE peptide was shown to be induced in plants by mechanical wounding and MeJA treatment, cleaved from the N-terminus of PR-1 proteins at a conserved CNYx sequence, and to activate the expression of proteinase inhibitor genes, PR-1, PR-7, and ERF-5 (Chen et al., 2014). The most highly expressed PR-1 genes in pea roots, Psat1g156240 and Psat1g213200, contain the CAPE peptide and the CNYx sequence (Fig. 1b) and the expression of Psat1g156240 was upregulated by MeJA at 24 h (Fig. 1c). This gene is used by researchers as an SA-dependent marker in pea leaves (Barba-Espín et al., 2011). Our results indicate that, at least in pea roots, this gene cannot be used to assess the activation of salicylate signaling. Conversely, the proteins encoded by Psat1g211480 and Psat0s4615g0040 contain a truncated CAPE peptide and lack the CNYx sequence. In pea roots, these genes are expressed at low levels and do not alter their expression in response to SA at the studied time points (Fig. 1a, b). It is possible that activation of these genes occurs earlier than 24 h. Activation of PR-1 genes by MeJA and other stimuli has been demonstrated previously. Activation of the AtPRB1 gene by JA/ethylene was shown in Arabidopsis roots (Santamaria et al., 2001). In rice, various PR-1 genes responded to SA and MeJA in a distinct manner, and organ‑specific variation in their expression levels was observed (Mitsuhara et al., 2008).

Chitinases (EC 3.2.1.14) belongs mainly to glycosyl hydrolase families 18 and 19, and degrade chitin, a cell walls component in many pathogenic microorganisms (Davies, Henrissat, 1995). Since chitinases were among the most significantly induced proteins in pea leaves under SA treatment (Tarchevsky et al., 2010), we analyzed expression of chitinase genes in roots. Furthermore, induction of chitinase transcripts by salicylate was demonstrated in pine (Davis et al., 2002).

According to transcriptomic data, the gene Psat5g000800 is highly expressed in pea roots, encodes a protein belonging to the GH19 family, and is characterized by the presence of a lysozyme-like domain. The expression of this gene is not sensitive to SA or MeJA (Fig. 2). It is suggested that GH19 chitinases play an important role in defense against soilborne pathogens and are secreted into the rhizosphere (De-la- Peña et al., 2010). The number of expressed chitinase genes in pea roots encoding GH18 proteins is greater compared to GH19, but their expression levels are lower than those of GH19 chitinase genes. Moreover, they do not show specificity in regulation by the studied phytohormones, except for Psat1g131280, the expression of which is significantly upregulated by MeJA (Fig. 2b). These data indicate that the chitinase isoforms expressed in pea roots cannot be used to assess the activation of the SA-dependent pathway.

Alongside genes encoding active chitinases, plant genomes contain genes encoding inactive chitinase-like proteins. During evolution, these proteins lost the ability to hydrolyze chitin due to amino acid substitutions in catalytically important domains and features of tertiary structure formation (Kesari et al., 2015). We identified significant activation of four chitinaselike protein genes in pea roots by SA (Fig. 3). Moreover, these genes were salicylate-dependent and not sensitive to MeJA. This makes them suitable candidates to use as markers of the SA-dependent pathway in pea roots, with the exception of Psat1g148600. We have shown that the content of the protein encoded by Psat1g148600 increases under action of the NO donor – sodium nitroprusside (Egorova, Tarchevsky, 2024).

Plant β-1,3-glucanases work in concert with chitinases, catalyzing the degradation of a microorganism’s cell wall components, β-1,3-glucans and chitin (Jongedijk et al., 1995; Dos Santos, Franco, 2023). The expression of β-1,3-glucanases and chitinases in pea roots is comparable and is represented by one highly expressed gene each. While some chitinase genes are induced by SA and MeJA, the β-1,3-glucanase encoded by Psat7g179640 is induced only by SA. The expression of other β-1,3-glucanase genes did not change under either SA or MeJA treatment

As mentioned earlier, Psat4g180800 encoding a PR-10 protein is the most highly expressed gene in pea roots, and its expression was not altered by SA or MeJA. Other genes encoding PR-10 proteins are largely insensitive to these phytohormones, and their expression remains unchanged under their influence.

One of the characteristics of PR proteins is their induction upon pathogen infection or under the action of stress phytohormones. Transcriptomic data analysis and our previous proteomic data indicate that in pea roots, the expression and content of some PR proteins are high – genes encoding certain PR proteins are among the most highly expressed genes in the control. These include the PR-1 gene Psat1g156240, and the PR-10 genes Psat4g180800 and Psat1g156920. This may be because roots are in constant contact with rhizosphere inhabitants, including pathogenic organisms. The existing pool of proteins with antipathogenic activity in roots allows them to be prepared for potential infection, and upon activation of the salicylate-dependent defense response, the expression and synthesis of chitinase-like proteins are activated, likely representing an additional defense mechanism in pea roots.

## Conclusion

The transcriptomic analysis of basal and inducible expression of certain PR genes in pea roots conducted in this work demonstrates that when selecting marker genes to assess the activation of salicylate signaling, attention to their specific expression patterns in the particular organ must be paid. Pea roots are characterized by a high level of expression of genes encoding antipathogenic proteins. Transcriptomic analysis revealed that the most highly expressed PR-1 genes in pea roots cannot be used to evaluate the activation of salicylate signaling. Chitinases and β-1,3-glucanases, induced by SA in pea leaves, did not show specificity in response to SA in roots. The obtained data indicate a difference in the response of leaves and roots to stress phytohormones. Using pea plants as an example, we showed that genes encoding salicylate-induced antipathogenic proteins in leaves are expressed at high levels in roots under normal conditions, while salicylate-induced genes in roots are those encoding chitinase-like proteins, which also have antipathogenic activity (unpublished data). In addition, the genes of chitinase-like proteins are practically not expressed in the control and are salicylate-inducible, which is an advantage when assessing the expression of these genes in order to evaluate the activation of the salicylate-dependent signaling pathway.

## Conflict of interest

The authors declare no conflict of interest.
